# Thyroglobulin Autoantibodies Do Not Influence the Outcome of Patients With Differentiated Thyroid Carcinoma

**DOI:** 10.1002/edm2.70198

**Published:** 2026-03-11

**Authors:** C. Bornemann, C. Bouter

**Affiliations:** ^1^ Department of Nuclear Medicine University Medical Center Göttingen (UMG), Georg‐August‐University Göttingen Germany

**Keywords:** follicular thyroid carcinoma, papillary thyroid carcinoma, thyroglobulin, thyroglobulin autoantibodies

## Abstract

**Introduction:**

Thyroglobulin (Tg) is a sensitive and specific marker for differentiated thyroid carcinoma (DTC). The presence of thyroglobulin autoantibodies (TgAb) can interfere with Tg measurement in commonly used immunoassays, often resulting in falsely low or undetectable Tg levels. However, data on the prognostic significance of TgAb or its potential utility as a surrogate marker for DTC are limited, leaving its role in the clinical management of DTC uncertain. The aim of this study was the evaluation of the prognostic significance of TgAb in a clinical cohort of patients with DTC.

**Methods:**

We included *n* = 289 patients with DTC who presented to our clinic for radioiodine treatment. Clinical data and follow‐up outcomes were retrospectively analysed. The TgAb status at initial treatment and during follow‐up was assessed, and its association with disease remission, persistence or recurrence was evaluated.

**Results:**

Roughly 25% of DTC patients in our cohort showed positive TgAb post‐surgery. There were no significant differences between TgAb‐positive and TgAb‐negative patients in histology, tumour size, metastasis, therapeutic regimen or risk stratification.

**Conclusion:**

The presence of TgAb did not influence the outcome regarding overall and disease‐free survival. Results implicate that the initial appearance of TgAb does not influence the outcome of patients with DTC. Patients with positive TgAb should be monitored closely during follow‐up as Tg measurement is unreliable, while overtreatment should be avoided.

## Graphical abstract

Thyroglobulin autoantibodies (TgAb) occur in roughly 25% of thyroid cancer patients and interfere with Tg measurement. Our study shows TgAb presence does not affect patient outcomes, but close monitoring is essential to avoid overtreatment.

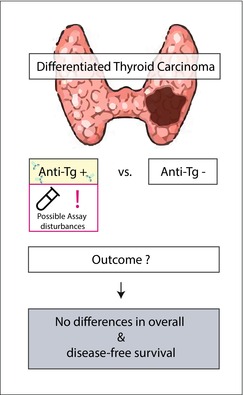

## 1 Introduction

Differentiated thyroid carcinoma (DTC) is a rare malignancy accounting for about 2% of all malignant diseases worldwide with rising incidences during the last decades [1, 2, 3]. Although survival rates surpass 98%, the occurrence of disease persistence and recurrence is reported to be as high as 30% [4, 5]. Follow up of DTC includes high resolution ultrasound of the neck and the measurement of the tumour marker thyroglobulin (Tg) in serum.

Tg is a sensitive and specific marker for DTC. However, thyroglobulin autoantibodies (TgAb) can interfere with the Tg measurement in commonly used immunoassays, leading to falsely low or undetectable Tg values. Positive TgAb display a significant clinical issue as they are detected in approximately 10% and 30% of patients with DTC [6, 7, 8, 9, 10, 11, 12]. Furthermore, interference with Tg measurement has implications for the risk assessment and outcome evaluation of DTC. Furthermore, changes in TgAb levels are suggested as a surrogate tumour marker in follow‐up. However, data on the prognostic significance of TgAb or its potential use as a surrogate tumour marker for DTC are limited and therefore its role in the clinical management of DTC remains unclear.

The aim of this study was the evaluation of the prognostic significance of TgAb in a clinical cohort of patients with DTC.

## 2 Material and Methods

### 2.1 Patients

We retrospectively evaluated *n* = 302 consecutive patients with DTC that presented in our clinic for radioiodine treatment between 2012 and 2023. All patients with histologically proven papillary or follicular carcinoma were included. Data on patient characteristics, history of thyroidectomy, use of postoperative radioiodine therapy, imaging results (radioiodine scan, ultrasound, and additional imaging using CT, MRI or FDG‐PET/CT), tumour characteristics, extension of disease, laboratory results (serum TSH, T3, T4, Tg and TgAb) and follow‐up data were obtained. *N* = 13 patients had to be excluded due to missing data, missing follow‐up or the use of a different Tg assay (Elecsys Tg II; Roche Diagnostics, Mannheim, Germany). *N* = 289 patients were included in the final analysis.

Remission, persistence or recurrence of disease was determined after thyroidectomy and radioiodine ablation. Remission was defined as no clinical, biochemical or structural evidence of disease after initial therapy (no detection of loco‐regional residual tumour tissue or metastasis in ultrasound and radioiodine whole‐body scan) and negative stimulated serum Tg and TgAb levels (TSH > 30 mIU/L after thyroid hormone withdrawal or rTSH stimulation).

Persistence or recurrence of disease were defined according to the American Thyroid Association Management Guidelines for Adult Patients with Thyroid Nodules and Differentiated Thyroid Cancer as: (1) structural: detection of residual tumour tissue or metastasis after thyroidectomy and radioiodine ablation or during follow‐up in imaging with ultrasound, whole‐body scan or in additional imaging procedures or detection of tumour tissue in fine‐needle aspiration cytology or histology, (2) biochemical: positive serum Tg (> 0.2 μg/L) without detection of residual tumour in imaging procedures, or (3) indeterminate: negative serum Tg and positive TgAb (> 14 IU/mL) without detection of residual tumour in imaging procedures [4].

TgAb status during initial treatment and during follow‐up was defined as negative or positive. Changes of TgAb levels compared to initial TgAb values were assessed in follow‐up and defined as follows: (1) stable, (2) increase > 50%, (3) decrease > 50%, (4) disappearing, or (5) de novo appearance.

### 2.2 Anti‐Thyroglobulin‐Antibody Measurement

TgAb were measured in clinical routine using the high sensitivity ARCHITECT Anti‐TG Assay, a chemiluminescent microparticle immunoassay calibrated against the WHO International Standard CRM 457 (Abbott Laboratories, Chicago, Illinois, USA). The validated analytical range was 1–1000 IU/mL. Values exceeding this range were obtained through manual dilution steps, if reported by the laboratory. Values ≥ 14 IU/mL were considered TgAb‐positive corresponding to the threshold defined by our institutional laboratory, and values < 14 IU/mL were considered TgAb‐negative throughout the study.

### 2.3 Thyroglobulin Measurement

Tg was measured in clinical routine using the high sensitivity ARCHITECT TG Assay (Abbott Laboratories, Chicago, Illinois, USA), calibrated against the WHO International Standard CRM 457. The analytical measurement range was 0.1–500 ng/mL; values exceeding this range were assessed following appropriate sample dilution, and values < 0.1 ng/mL were considered Tg‐negative as defined by our institutional laboratory.

### 2.4 Statistical Analysis

Statistical analysis was performed using GraphPad Prism version 9 (GraphPad Software, San Diego, CA, USA) and SPSS Statistics version 28 (IBM, Armonk, NY, USA). Descriptive statistics were performed on all collected variables. Differences between groups were tested using the Mann–Whitney test. The Chi‐square test was used for categorical variables. The Log‐rank test was used to compare the probabilities of occurrence of recurrence and survival between groups. To assess the independent effect of TgAb status on recurrence‐free survival while adjusting for potential confounders, a multivariate Cox proportional hazards regression analysis was performed. Logistic regression was used to test for the dependence of the patient's outcome on tested variables.

Data are presented as mean ± standard deviation (SD) or median with 25th percentile (Q1), 75th percentile (Q3) and interquartile range (IQR). Significance levels are given as follows: *p < 0.05; **p < 0.01; ***p < 0.0001.

The study was approved by the local ethical committee of the UMG Goettingen and written informed consent has been obtained from each patient after full explanation of the purpose and nature of all procedures used.

## 3 Results

### 3.1 Patient Characteristics

A total of *n* = 289 patients were included in this study. Patient characteristics are shown in Table 1. *N* = 178 patients were female (61.6%) and *n* = 111 were male (38.4%). The mean age at diagnosis was 50.1 ± 16.9 years. All patients underwent thyroidectomy with or without lymph node dissection and at least one radioiodine treatment. Papillary thyroid carcinoma was detected in *n* = 234 patients (81%) and follicular thyroid carcinoma in *n* = 55 patients (19%). Mean stimulated Tg levels (TSH > 30 mIU/L) were 143.8 ± 641.8 μg/L at the first radioiodine treatment.

**TABLE 1:** Patient characteristics.

	TgAb+ (*n* = 69)	TgAb− (*n* = 220)	*p*
Age (years, mean ± SD)	47.1 ± 17.5	50.4 ± 16.5	0.1574
Gender			
Female (*n*)	50 (72.5%)	128 (58.2%)	0.0344
Male (*n*)	19 (27.5%)	92 (41.8%)	
Histology			
Papillary (*n*)	60 (87%)	174 (79.1%)	0.1464
Follicular (*n*)	9 (13%)	46 (20.9%)	
Multifocality (*n*)	7 (10%)	17 (7.7%)	0.5255
TNM‐classification (*n*)			
T1	38 (55.1%)	98 (44.5%)	0.3861
T2	12 (17.4%)	53 (24%)	
T3	18 (26.1%)	56 (25.4%)	
T4	1 (1.4%)	8 (3.6%)	
TX	0 (0%)	5 (2.3%)	
N0	46 (66.7%)	151 (68.6%)	0.7686
N1	23 (33.3%)	69 (31.4%)	
M0	62 (89.8%)	200 (90.9%)	0.8137
M1	7 (10.2%)	20 (9.1%)	
ATA risk stratification (*n*)			
Low	45 (65.2%)	126 (57.3%)	0.4978
Intermediate	7 (10.1%)	29 (13.2%)	
High	17 (24.6%)	65 (29.5%)	
History of autoimmune disorder (*n*)	19 (27.5%)	19 (8.6%)	*p* < 0.0001
Radioiodine treatment			
Mean activity of I‐131 (GBq) at ablation	5.575 ± 2.8	6.055 ± 2.8	0.1977
Mean number of radioiodine therapies	2 ± 0.8	2 ± 0.8	0.9388
Mean total activity of I‐131 (GBq)	9.366 ± 5.2	10.05 ± 5.9	0.5733
Additional diagnostics using 18F‐FDG‐PET			
18F‐FDG‐PET at first radioiodine treatment (*n*)	8 (11.6%)	40 (18.2%)	0.2658
18F‐FDG‐PET during follow‐up	7 (10.1%)	17 (7.7%)	0.6167

### 3.2 Thyroglobulin Autoantibodies

**FIGURE 1:**
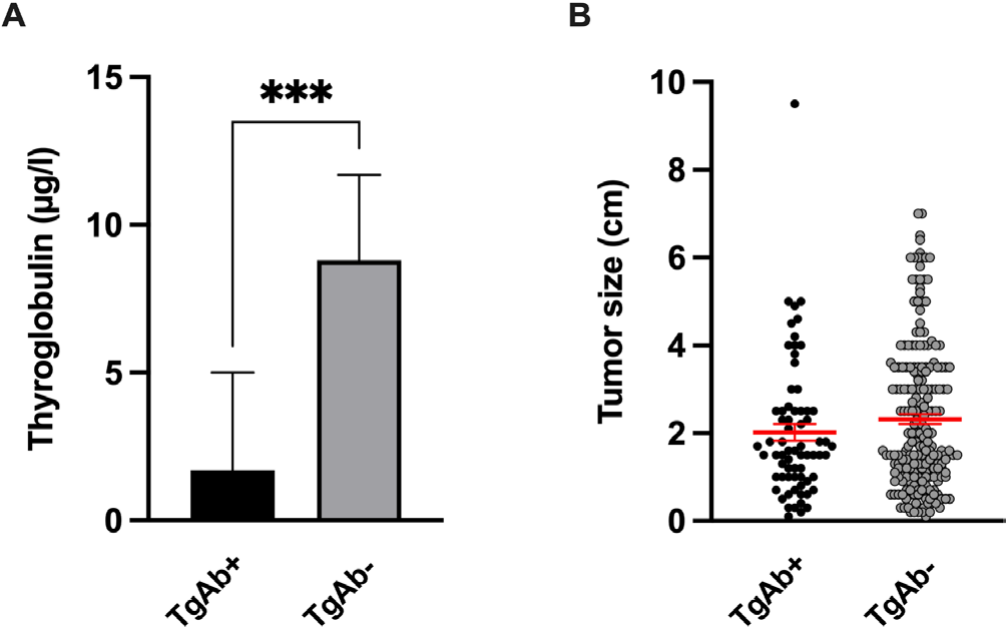
Tumour size and initial thyroglobulin levels. (A) Initial postoperative stimulated thyroglobulin levels (TSH levels > 30 mIU/L) were significantly lower in TgAb‐positive patients compared to TgAb‐negative patients (***p < 0.0001; Mann–Whitney test). (B) Tumour size did not show significant differences between TgAb‐positive and TgAb‐negative patients (*p* = 0.1798; Mann–Whitney test).

At the time of the initial diagnosis *n* = 69 patients (23.9%) showed positive TgAb (Median TgAb level: 51.0 IU/mL; 25th percentile (Q1): 26.0 IU/mL; 75th percentile (Q3): 172.5 IU/mL; IQR: 146.5 IU/mL), while the remaining *n* = 220 patients (76.1%) showed negative TgAb‐levels. Initial stimulated Tg‐levels were significantly higher in TgAb‐negative patients compared to TgAb‐positive patients (*p* < 0.0001; Mann–Whitney test; Figure 1A). *N* = 25 cases (36.2%) of the TgAb‐positive group showed negative Tg during the initial radioiodine therapy, although thyroid remnants (*n* = 25) and metastases (*n* = 1) were detectable on radioiodine imaging and ultrasound.

The TgAb‐positive group showed significantly more female patients compared to the TgAb‐negative group (*p* = 0.0344; Chi‐square test). There were no significant differences between TgAb‐positive and TgAb‐negative patients in the age at the time of the diagnosis (*p* = 0.1574; Mann–Whitney test), histologic subtypes (*p* = 0.1464; Chi‐square test), tumour size (*p* = 0.1798; Mann–Whitney test; Figure 1B), multifocality (*p* = 0.5255; Chi‐square test), TNM classification (T: *p* = 0.3861; N: *p* = 0.7686; M: *p* = 0.8137; Chi‐square test) or ATA risk stratification (*p* = 0.4978; Chi‐square test). The prevalence of previously diagnosed autoimmune thyroid disorders was significantly higher in the TgAb‐positive group compared to the TgAb‐negative group (*p* < 0.0001, Chi‐square test; Table 1).

### 3.3 Radioiodine Treatment and Additional Diagnostics

TgAb‐positive and TgAb‐negative groups did not show differences in the used radioiodine activity at ablation (*p* = 0.1977), mean number of radioiodine therapies (*p* = 0.9388), and mean total administered radioiodine activity (*p* = 0.5733; Mann–Whitney test; Table 1). Furthermore, the use of 18F‐FDG‐PET during the first radioiodine treatment (*p* = 0.2658) or during follow‐up (*p* = 0.6167; Chi‐square test; Table 1) did not show differences between the TgAb‐positive and TgAb‐negative patients, either.

### 3.4 Initial Response After Primary Therapy

After thyroidectomy and radioiodine ablation, *n* = 144 (49.8%) patients achieved remission, while *n* = 145 (50.2%) showed persistent disease. The disease status did not show significant differences between TgAb‐positive and TgAb‐negative patients (Chi‐square test: *p* = 0.0783; Table 2). Among the patients with persistent disease, the type of persistence (structural, biochemical, indeterminate) differed significantly between TgAb‐positive and TgAb‐negative patients, with a higher frequency of structural disease in the TgAb‐negative group (*p* = 0.0002, Table 2).

**TABLE 2:** Response after primary treatment and last available follow‐up.

	TgAb+ (*n* = 69)	TgAb− (*n* = 220)	*p*
Initial response after primary therapy			0.0783
Remission	28 (40.6%)	116 (52.7%)	
Persistence	41 (59.4%)	104 (47.3%)	0.0002
Structural	17 (41.5%)	63 (60.6%)	
Biochemical	18 (43.9%)	41 (39.4%)	
Indeterminate	6 (14.6%)	—	
Response at last follow‐up			0.1830
Remission	45 (65.2%)	146 (66.4%)	
Persistence	20 (29%)	65 (29.5%)	
Structural	6	31	
Biochemical	6	33	
Indeterminate	8	1	
Disease‐related death	4 (5.8%)	9 (4.1%)	

Abbreviation: TgAb, anti‐thyroglobulin antibodies.

### 3.5 Follow Up and Secondary Remission Following Further Therapy

The mean follow‐up period was 40.4 ± 28.5 months. At last follow‐up, *n* = 191 (66.1%) patients were disease‐free, *n* = 85 (29.4%) had persistent disease, and *n* = 13 (4.5%) had died of the disease (metastasis affecting multiple organs: *n* = 5; lung metastasis: *n* = 8). The disease status at the last follow‐up did not show significant differences between TgAb‐positive and TgAb‐negative patients (Chi‐square test: *p* = 0.1830; Table 2). Among the patients with persistent disease, the type of persistence (structural, biochemical, indeterminate) differed significantly between TgAb‐positive and TgAb‐negative patients with a higher frequency of structural disease in the TgAb‐negative group (*p* = < 0.0001, Table 2).

Among patients with initial disease persistence, *n* = 48 (33%) achieved remission after a mean of 30 ± 23.7 months. *n* = 19 (39.5%) of them were initially TgAb‐positive. Remission was achieved through surgery (*n* = 5), additional radioiodine therapy (*n* = 29), or confirmed during follow‐up by negative stimulated Tg/TgAb and imaging (*n* = 14). Recurrence after initial remission recurred in *n* = 2 cases, both with initially negative TgAb.

### 3.6 Impact of TgAb Status on Outcome

**FIGURE 2:**
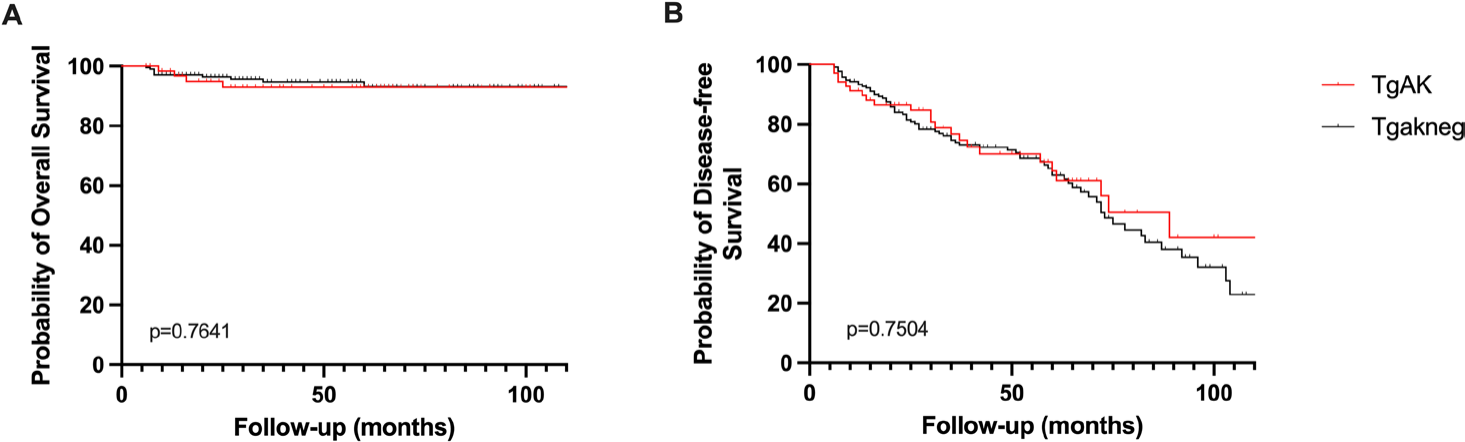
Overall and disease‐free survival. (A) Probability of overall survival did not show significant differences between TgAb‐positive and TgAb‐negative patients (*p* = 0.7641; Log‐rank test). TgAb status was not significantly associated with overall survival (HR = 1.394; 95% CI: 0.403–4.825; *p* = 0.6; multivariate Cox regression) after adjusting for confounders. (B) Probability of disease‐free survival did not show significant differences between TgAb‐positive and TgAb‐negative patients (*p* = 0.7508; Log‐rank test). TgAb status was not significantly associated with risk of recurrence (HR = 1.046; 95% CI: 0.618–1.77; *p* = 0.867; multivariate Cox regression) after adjusting for confounders.

The probability of overall survival did not show significant differences between TgAb‐positive and TgAb‐negative patients (*p* = 0.7641; Log‐rank test; Figure 2A). Similarly, in the multivariate Cox regression model, TgAb status was not significantly associated with overall survival (HR = 1.394; 95% CI: 0.403–4.825; *p* = 0.6) after adjusting for histological subtype, tumour size, history of autoimmune disorder, ATA risk, number of radioiodine therapies and administered radioiodine activity.

Probability of disease‐free survival did not show significant differences between TgAb‐positive and TgAb‐negative patients (*p* = 0.7508; Log‐rank test; Figure 2B). TgAb status was not significantly associated with risk of recurrence (HR = 1.046; 95% CI: 0.618–1.77; *p* = 0.867; multivariate Cox regression) after adjusting for histological subtype, tumour size, history of autoimmune disorder, ATA risk, number of radioiodine therapies and administered radioiodine activity.

### 3.7 Predictors

Furthermore, predictors of the patient's outcome were evaluated. The presence of distant metastasis was the strongest risk factor for disease persistence (OR = 15.709; 95% CI: 5.285–46.692; *p* < 0.0001), while the appearance of TgAb did not show an association with disease persistence (OR = 1.03; 95% CI: 0.584–1.82; *p* = 0.916; univariate logistic regression; Table 3).

**TABLE 3:** Risk factors for disease persistence.

Factor	OR (95% CI)	*p*
Age	1.023 (1.008–1.039)	0.003
Gender	0.434 (0.264–0.715)	0.001
Histological subtype	0.506 (0.278–0.919)	0.025
Tumour size	1.525 (1.294–1.796)	< 0.0001
Multifocality	1.724 (0.888–3.348)	0.107
Lymph node metastasis	3.42 (2.025–5.778)	< 0.0001
Distant metastasis	15.709 (5.285–46.692)	< 0.0001
Positive TgAb	1.031 (0.584–1.82)	0.916
Initial Tg value	1.008 (1.004–1.012)	< 0.0001
History of autoimmune disorder	0.755 (0.357–1.594)	0.46
ATA risk stratification		
Low	0.175 (0.103–0.298)	< 0.0001
Medium	2.056 (0.929–4.552)	0.075
High	7.146 (3.965–12.879)	< 0.0001

Abbreviations: ATA, American Thyroid Association; Tg, thyroglobulin; TgAb, anti‐thyroglobulin antibodies.

### 3.8 TgAb Trend and Clinical Outcome in Follow Up

Data on TgAb trends in follow‐up are shown in Table 4. Among *n* = 69 patients with initially positive TgAb, *n* = 6 showed stable TgAb levels during follow‐up. The median TgAb level was 332.5 IU/mL (25th percentile: 75.25 IU/mL; 75th percentile: 1434 IU/mL; IQR: 1358.75 IU/mL). *N* = 9 patients showed a decrease in TgAb levels of more than 50% compared to baseline. The median TgAb level was 25.0 IU/mL (25th percentile: 21.5 IU/mL; 75th percentile: 62.0 IU/mL; IQR: 40.5 IU/mL). The remaining *n* = 54 cases showed negative TgAb levels during follow‐up. None of the patients showed increasing TgAb levels at follow‐up. The clinical outcome did not show significant differences between patients with stable, decreasing or disappearing TgAb levels (Chi‐square test: *p* = 0.2574; Table 4).

**TABLE 4:** Trends in TgAb levels during follow‐up and their association with structural disease.

	Total	Stable TgAb	Disappearing TgAb	Decreasing TgAb > 50%	Increasing TgAb > 50%	*p*
TgAb positive	69	6	54	9	—	0.2574
Structural persistence—local	3	3	—	2	—	
Structural persistence—distant	5	1	3	1	—	
Local recurrence	4	—	1	—	—	
No signs of structural disease	58	2	50	6	—	

Abbreviation: TgAb, anti‐thyroglobulin antibodies.

### 3.9 De Novo Appearance of TgAb

Among *n* = 220 patients with initially negative TgAb, *n* = 5 patients showed a transient de novo appearance of TgAb after initial therapy. While *n* = 1 of these patients showed a de novo appearance of TgAb that persisted until the last follow‐up without any sign of structural disease. All cases showed persistent disease until the last available follow‐up (structural: *n* = 3; biochemical: *n* = 3; and indeterminate: *n* = 1). There were no differences in the type of persistence between the group of patients with a de novo appearance of TgAb compared to the group of patients that remained TgAb‐negative throughout the study (*p* = 0.1157).

## 4 Discussion

The detection of TgAb during initial treatment or follow‐up of patients with DTC is a known clinical issue as TgAb can interfere with Tg assays, resulting in falsely low or undetectable Tg values. As Tg measurement is inaccurate, DTC patients with positive TgAb are monitored closely in follow‐up presentations using neck ultrasound or radioiodine‐ or PET‐imaging. However, only a limited number of studies on the prognostic impact of TgAb are available and the clinical significance of positive TgAb in DTC remains unclear. Therefore, the aim of our study was the evaluation of the prognostic significance of TgAb in a clinical cohort of patients with DTC.

Current ATA‐guidelines recommend the use of TgAb levels over time as a useful parameter for the determination of the risk of disease recurrence. Therefore, TgAb are included in the definition of the response after therapy. Rising TgAb are defined as a sign of biochemical incomplete response and stable or declining TgAb levels are of indeterminate response [4].

### 4.1 TgAb+ Initially

Roughly 25% of DTC patients in our cohort showed positive TgAb post‐surgery. The incidence of TgAb was comparable to previous studies, which showed a prevalence of TgAb in patients with DTC ranging between 10% and 30% [6, 7, 8, 9, 10, 11, 12]. Our cohort did not show significant differences between TgAb‐positive and TgAb‐negative patients in the histological subtypes, tumour size, multifocality, TNM‐stage or ATA risk stratification. Data is in line with several earlier studies, which did not detect differences between tumour size or distant metastasis, either [13, 14, 15, 16]. Furthermore, postoperative TgAb status did not influence the prognosis of DTC patients in our cohort. Neither overall survival, nor disease‐free survival was affected by initial TgAb appearance. Our data is in line with a previous study by McLeod et al. [17] that showed that initially TgAb do not predict the outcome of thyroid carcinoma in a multicenter register of DTC patients in North America.

### 4.2 TgAb As Surrogate Tumour Marker

A small number of patients in our cohort (*n* = 6) showed a de novo appearance of TgAb in follow‐up, while none of the TgAb‐positive cases showed increasing TgAb levels. Patients with a de novo appearance of TgAb showed disease persistence with metastatic disease or complete remission in further follow‐up, but no disease progression was detected. The management of these patients was changed due to the de novo appearance of TgAb with a closer follow‐up or the addition of I‐131 or 18F‐FDG‐PET diagnostics. The guidelines do not provide a standardised protocol for further diagnostics in these cases.

In our study, a transient de novo appearance of TgAb was observed in six patients, all of whom demonstrated biochemical or structural persistence, but no documented recurrence following complete remission. Our data is in line with earlier studies that could not show a clinical impact of a transient de novo appearance of TgAb in the follow up of patients with differentiated thyroid carcinoma, either [18, 19]. The clinical relevance of such TgAb dynamics remains unclear. One possible explanation is that these fluctuations reflect a reactivation of the immune response to residual thyroglobulin or tumour tissue. Alternatively, the TgAb rise could be triggered by antigen release from structurally stable but metabolically active remnants. However, it is also important to consider analytical variability as a potential cause. Although minor fluctuations in TgAb levels may occur near the lower detection limit due to assay imprecision, the TgAb values observed in these six patients were well above typical analytical noise and demonstrated a clear kinetic pattern. Therefore, we believe these changes are more likely to represent biologically meaningful immunological responses rather than random assay variation. Further prospective studies with standardised assay methods and serial immunological profiling will be required to distinguish true immunological TgAb kinetics from potential laboratory artefacts and to better define their prognostic significance.

Furthermore, TgAb were not useful as a surrogate tumour marker in our cohort. However, subgroups are statistically underpowered and might not be able to detect clinically meaningful associations. A few studies were able to show a connection between TgAb‐levels and the risk of disease recurrence and therefore sustained de novo appearance of TgAb or rising TgAb‐levels might predict structural recurrence and be possibly useful as a surrogate tumour marker [14, 20, 21, 22]. Overall, rising TgAb‐levels or the de novo appearance of TgAb should be taken seriously and possible disease recurrence or progress should be evaluated. Patients should be monitored closely, while overtreatment should be avoided in cases with decreasing or stable TgAb‐levels.

### 4.3 Autoimmune Disease

The prevalence of TgAb is higher in DTC patients compared to the general population. As described above, the prevalence of TgAb was 25% in our cohort, while the prevalence of TgAb in the general population varies across studies and populations with a reported prevalence of TgAb ranging from 4% to 16% [6, 23]. TgAb are often detected in patients with autoimmune thyroid disease. In our cohort, 14% of DTC patients had a history of autoimmune thyroid disease, and 50% of these patients showed positive TgAb. Overall and disease‐free survival did not show significant differences between patients with an autoimmune thyroid disorder compared to patients with no history of autoimmune thyroid disease. However, thyroid autoimmunity is suggested as a risk factor for the development of thyroid carcinoma probably due to chronic inflammatory processes in the thyroid [24, 25, 26].

### 4.4 Clinical Implications

The presence of TgAb did not influence the outcome regarding overall and disease‐free survival in our cohort. Administered activity at radioiodine treatment for ablation did not differ between TgAb‐positive and TgAb‐negative groups either. In view of all available data, an aggressive therapeutic and diagnostic approach on patients with DTC and positive TgAb does not appear necessary. However, TgAb should be monitored routinely in the follow‐up of DTC patients in order to detect possible assay interferences that lead to false negative Tg values, which is already recommended in clinical guidelines [4]. In our study, 36% of patients with positive TgAb showed a negative Tg, although thyroid remnants or metastases were detectable on imaging. This discrepancy is explained by antibody interference in Tg measurement. Therefore, it is necessary to monitor TgAb levels, especially in patients with negative Tg that do not match other findings as Tg becomes unreliable as a tumour marker in the presence of TgAb.

### 4.5 Limitations

Limitations of the study include the retrospective setting. Due to this study design, the data collection relied on the patients' medical records and clinical examinations without a standardised protocol, especially the use of diagnostics as 18F‐FDG‐PET was decided on a single case level without the use of a standardised protocol which could lead to possible selection bias. The cohort predominantly comprised low‐risk patients (59% low‐, 13% intermediate‐, 28% high‐risk) reflecting the typical epidemiological profile in our clinic. This may underestimate the prognostic role of TgAb in high‐risk populations. Furthermore, we did not include patients with microcarcinomas that did not undergo radioiodine treatment. Another limitation is the small number of patients in some TgAb‐positive subgroups, especially those with stable or increasing TgAb and those with newly developed TgAb positivity. Another major limitation of our study is the relatively short median follow‐up time of 40.9 months, which may be insufficient to capture late recurrences or long‐term survival differences, particularly in intermediate‐ and high‐risk patients. Therefore, the prognostic implications of TgAb dynamics over longer periods require further investigation in extended follow‐up cohorts. In order to overcome these limitations prospective studies in larger cohorts are needed.

## 5 Conclusion

Data from our study implicates that the initial appearance of TgAb does not influence the outcome of patients with DTC. Patients with positive TgAb should be monitored closely during follow‐up as Tg measurement is unreliable. However, overtreatment should be avoided.

## Author Contributions

**C. Bornemann:** performed data collection and analyzed data. **C. Bouter:** conceived the study, analyzed data, interpreted results and wrote the manuscript.

## Funding

The authors have nothing to report.

## Conflicts of Interest

The authors declare no conflicts of interest.

## Data Availability

The data that support the findings of this study are available on request from the corresponding author. The data are not publicly available due to privacy or ethical restrictions.
